# Enrolling pregnant women in research: ethical challenges encountered in Lao PDR (Laos)

**DOI:** 10.1186/s12978-017-0428-9

**Published:** 2017-12-14

**Authors:** Vilada Chansamouth, Rose McGready, Danoy Chommanam, Soukanya Homsombath, Mayfong Mayxay, Paul N. Newton

**Affiliations:** 1 Lao-Oxford-Mahosot Hospital-Wellcome Trust Research Unit (LOMWRU), Microbiology Laboratory, Mahosot Hospital, Vientiane, Lao People’s Democratic Republic; 2 Shoklo Malaria Research Unit (SMRU), Mahidol-Oxford Tropical Medicine Research Unit, Mae Sot, Tak, Thailand; 3 Pakngum District Hospital, Vientiane, Lao People’s Democratic Republic; 4 Nuffield Department of Medicine Research Building, Centre for Tropical Medicine and Global Health, University of Oxford, Oxford, England, UK; 5 Faculty of Postgraduate Studies, University of Health Sciences, Vientiane, Lao People’s Democratic Republic

**Keywords:** Lao, Pregnancy, Mortality, Cohort, Culture, Decision-making, Misconception, Infection

## Abstract

Laos has the highest maternal mortality ratio in mainland Southeast Asia but there has been little research conducted with pregnant women. We aim to discuss ethical challenges in enrolling pregnant women in research as a part of large pregnancy cohort study in Laos. From 2013 to 2015, a prospective cohort study was conducted with 1000 pregnant women in a rural area of Vientiane, Laos, to determine whether fevers were associated with maternal morbidity and small for gestational age. Incidence of fever was 10% and incidence of small for gestational age was 12%. Level of education, cultural norms about family decision-making, and misconceptions about healthcare during pregnancy were three common issues encountered in enrolling pregnant women to this study. Only 47% of recruited women had completed primary school with no further education, which could affect the decisions women make to participate and remain in the study. Family decision-making is common in Laos; in some cases, we could not recruit pregnant women without agreement from their families. In Laos, many pregnant women and their families had strong beliefs in travelling during late pregnancy or losing small amount of blood (giving ~5 ml blood sample) could negatively impact their pregnancies. These misconceptions affected not only the quality of the study but also the women’s opportunities to access healthcare. Good engagement between the research team and study participants, and the provision of more health information to the community, were essential to reducing issues experienced in enrolling pregnant women in this study.

## Case background

Laos is a lower middle-income country in Southeast Asia (SEA) [1] with the highest maternal mortality ratio in mainland SEA (197/100,000 live births) [2] and a high incidence of infectious diseases [3, 4]. There has been little research conducted with pregnant women to inform policy to reduce their mortality in Laos. There has been no population-based study of febrile illnesses in pregnant Lao women. Data on the causes and consequences of febrile illnesses in pregnant women will provide information to guide the future care of such women, especially in remote areas where diagnostic tools are limited. This article aims to discuss ethical challenges in enrolling pregnant women in research.

From 2013 to 2015, a community-based, prospective cohort study was conducted to investigate whether fevers were associated with maternal morbidity and small for gestational age (SGA) in Pakngum District, Vientiane City, Laos. We recruited 1000 pregnant women of any age and any gestational stage during the 18-month recruitment period. All women who consented were followed up every 2 months until 6 weeks postpartum. On recruitment, consenting women were asked for 10 ml of blood, urine, and nasopharyngeal swabs as baseline samples. Screening for syphilis, full blood count testing, and obstetrical ultrasound were performed without charge. During the follow-up period, any participants who developed a fever were offered admission to hospital. Causes of fever were investigated using serology tests, PCR assays, and conventional blood culture, which were additional investigations provided by the study. Febrile women received free healthcare during their admission. Treatment was based on laboratory investigations, but empirical therapy was given as thought appropriate by the responsible physician. Any medication given to pregnant women, including empirical antibiotic treatment, was carefully chosen according to pregnancy guidelines. At birth, placenta, cord blood, and blood samples from participant women were collected. Lao government provides free healthcare for all children under age five in Laos [5]. Another study and local doctors would manage and evaluate sick newborn babies and refer to central hospitals, if necessary.

Of the 1084 pregnant women screened, 47 (4%) did not meet the inclusion criteria and 37 (3%) declined to participate; of the 1000 pregnant women recruited, 15 (2%) were lost to follow-up. The frequency of fever in pregnant women over the course of 25 months was 10%. The most common cause of fever was influenza, followed by pyelonephritis and rickettsial diseases. The results of the study demonstrated no evidence of association between SGA rates and maternal fever (*p* = 0.99). Miscarriage, stillbirth, maternal and neonatal deaths, and congenital abnormality were also assessed in the study.

## Ethical discussion

The common issues that we encountered in recruiting Lao pregnant women were related to (1) level of education; (2) cultural norms about family decision-making; and (3) mistaken beliefs about research procedures and medical care.

1. According to the Lao Social Indicator Survey 2011–2012, only 69% of Lao women between 15 and 24 years of age are literate [6]. In our study, 47% of recruited women had completed primary school with no further education. Though we did not examine health understanding of our study participants, pregnant women’s level of education could affect their decisions to participate in the study, to adhere to the study’s schedule for follow-up, and to withdraw from the study.
2. Lao women are influenced by their husbands and mothers in terms of their behavior during pregnancy and lactation [7]. This is a sensitive situation in Lao society. In our study, nearly one-third of women who declined to consent did so because her family refused, even though the pregnant woman herself wanted to participate. For example, one pregnant woman wanted to enroll in the study, but her mother-in-law did not allow her to participate. The mother-in-law said that participation in the study would waste time that could be spent taking care of the family and doing household work; the pregnant woman ultimately chose not to enroll in the study. If their relatives did not agree, we had to accept that these women would not participate in order to avoid arguments within their family. However, our study team offered health information to these women and the opportunity to discuss health issues during pregnancy if they wished. These women could still participate in the study at any time during their pregnancies if their family members changed their minds.
3. Some pregnant women believed that traveling during late pregnancy could negatively impact their pregnancies. Additionally, some women refused to come for a routine follow-up or even to hospital when they were ill. Though blood samples for diagnostic assays in pregnant women with fever—as required in our study—are highly beneficial to obtaining a diagnosis, some pregnant women and their families had strong opinions about giving blood during pregnancy. Some believed that even a small volume (~5 ml) blood sample could affect the health of the woman and the future child; these women decided not to give additional diagnostic samples later in the study. This affected not only the quality of the study to diagnose some pathogens, but also the healthcare provided to the women. Some refused to receive further diagnostic investigation or care and asked to leave the hospital even if they were ill. This unwillingness to receive diagnosis or treatment posed an ethical challenge: in this study, febrile pregnant women received access to full, free care and treatment, which was superior to standard care normally available to them. Respecting the right of the patient to refuse treatment, even when the patient (or her family) refuses to consent to treatment due to misconceptions, could result in preventable maternal or neonatal death.

Some misconceptions circulate in society for a long time and are not easy to change. Providing health information and education to the entire community is vital in this situation. Most of the time, training and encouraging members of the community to talk with their community members gives favorable results. Our study team, with the help from the local public health staff, explained what benefits the women would receive from further investigations or returning to hospital. Many of the women also agreed to let our team and local public health staff visit them at home after explaining the benefits of study participation.

## Conclusions

Good engagement between the research team and study participants is key to reducing the ethical issues discussed. The research team should be trained on how to approach pregnant women and provide as much information as possible to ensure that the women understand the aims and methods of the projects. In addition, in this setting it is important that the woman’s family also understand and support her. For example, some research team members may have been too rushed to enroll study participants without clearly explaining the relevant study processes. Women sometimes found out later that they were going to be asked to give more samples, which could have led to their withdrawal from the study.

Giving more health information to the community will improve both educational issues and reduce traditional beliefs that modern public health suggests are harmful. The research team must provide correct information not only to pregnant women, but also to their husbands and other family members. Moreover, the research team must respect the women’s decisions. Understanding cultural barriers to particular research projects and associated appropriate clinical care in specific settings is also vital.

## Abbreviations

SEA: Southeast Asia
SGA: Small for gestational age
